# Microwave Imaging System Based on Signal Analysis in a Planar Environment for Detection of Abdominal Aortic Aneurysms

**DOI:** 10.3390/bios14030149

**Published:** 2024-03-18

**Authors:** Andrea Martínez-Lozano, Roberto Gutierrez, Carlos G. Juan, Carolina Blanco-Angulo, Héctor García-Martínez, Germán Torregrosa, José María Sabater-Navarro, Ernesto Ávila-Navarro

**Affiliations:** 1 Microwave Laboratory Research Group, Engineering Research Institute of Elche, Miguel Hernández University of Elche, 03202 Elche, Spaincarolina.blanco01@goumh.umh.es (C.B.-A.); mhector@umh.es (H.G.-M.); gtorregrosa@umh.es (G.T.); eavila@umh.es (E.Á.-N.); 2 Neuroengineering Biomedical Research Group, Institute of Bioengineering, Miguel Hernández University of Elche, 03202 Elche, Spain; 3 Electronic Design and Signal Processing Techniques Research Group, Department of Electronics, Computer Technology and Projects, Technical University of Cartagena, 30202 Cartagena, Spain

**Keywords:** abdominal aortic aneurysm (AAA), Delay-and-Sum (DAS), Improved Delay-and-Sum (IDAS), microwave medical imaging (MWI), non-invasive measurement, radio-frequency antenna system

## Abstract

A proof-of-concept of a microwave imaging system for the fast detection of abdominal aortic aneurysms is shown. This experimental technology seeks to overcome the factors hampering the fast screening for these aneurysms with the usual equipment, such as high cost, long-time operation or hazardous exposure to chemical substances. The hardware system is composed of 16 twin antennas mastered by a microcontroller through a switching network, which connects the antennas to the measurement instrument for sequential measurement. The software system is run by a computer, mastering the whole system, automatizing the measurement process and running the signal processing and medical image generation algorithms. Two image generation algorithms are tested: Delay-and-Sum (DAS) and Improved Delay-and-Sum (IDAS). Own-modified versions of these algorithms adapted to the requirements of our system are proposed. The system is carefully calibrated and fine-tuned with known objects placed at known distances. An experimental proof-of-concept is shown with a human torso phantom, including an aorta phantom and an aneurysm phantom placed in different positions. The results show good imaging capabilities with the potential for detecting and locating possible abdominal aortic aneurysms and reporting acceptable errors.

## 1. Introduction

An aneurysm is a permanent, irreversible, localized dilation in a blood vessel [1]. Aneurysms may appear everywhere in the body, although they are more likely to be found in certain regions such as the lower limbs, head, neck, abdomen or thorax [2]. Aneurysms happen in individuals of all ages, with a similar incidence for both genders. Due to the difficulty of early detection, they may yield to severe problems or even, if the right treatment is not applied, to death [3]. The severity of an aneurysm is directly related to its location and size. The ones appearing in the neck and upper limbs are usually asymptomatic, only receiving treatment due to aesthetic issues. The aneurysms in the thorax and abdomen are usually asymptomatic as well. However, unlike thoracic ones, abdominal aneurysms show a higher probability of developing further complications derived from vessel dilation, such as gastrointestinal hemorrhages or thromboembolisms. In the lower limbs, the most common ones are the popliteal aneurysms (in the back of the knee), often associated with patients suffering thromboembolic illnesses or chronic venous insufficiency. Currently, there is deep knowledge of the possible complications derived from aneurysms, given that it is a common and widespread health problem, although the knowledge about the origin and development of this issue is still limited [4].

Among the different kinds of aneurysms, one of the most important ones, due to its high mortality rate, is the abdominal aortic aneurysm (AAA). An AAA is diagnosed when, due to weak abdominal aorta boundaries, the aorta is wider than usual, reaching an increment of at least 50% of its normal diameter [5,6]. In general, AAA is diagnosed when the diameter of the abdominal aorta surpasses 30 mm [1]. On average, an AAA grows between 0.3 and 0.4 cm per year [7]. This kind of aneurysm is one of the most common aortic diseases [8]. It is more likely to appear in aged people, and it may lead to death if proper treatment is not applied [9]. The risk factors associated with AAA include being more than 75 years old, male, Caucasian, a smoker, having prior vascular diseases, and having wrongly controlled hypertension and hypercholesterolemia [5,9]. Indeed, AAA is directly associated with smoking and cerebrovascular diseases [9]. The risk of rupture of the aorta due to an AAA is proportional to the size of the aneurysm, its growing trend, the thickness of the boundaries of the aorta and the biomechanical stress found in the area of the aneurysm [5,9].

Due to all these reasons and potential hazardous complications, the early and effective detection of aneurysms is vital. Within the usual diagnosis methods used for screening AAA, we can find the evaluation of the pulsatile mass, performed with two-hand palpation in the supraumbilical area, which means that the bigger the AAA, the more sensitive this technique is [1], which is not convenient for early detection. More sophisticated methods make use of medical imaging techniques, such as radiography, echography, computerized tomography (CT) or magnetic resonance (MR). The most common method for AAA assessment and tracking is echography. This is a cost-effective and simple medical imaging method that allows medical practitioners to evaluate the diameter of the aorta with acceptable precision and in a non-invasive manner. When an aneurysm shows a considerable size, and it may potentially lead to severe complications, the patient must undergo surgery. In this case, it is more common to use CT or MR to obtain high-resolution vascular images. This allows the doctors to reach a precise diagnosis and a better preoperative evaluation devoted to selecting the most appropriate treatment, usually endovascular or open surgery [1,6,10].

The use of medical imaging techniques for AAA diagnosis is wide nowadays. Notwithstanding that, these techniques still show certain drawbacks limiting their use. Echography is limited by a low contrast and low resolution for the aorta images [10,11]. CT requires the patient to be exposed to both ionizing radiation and chemical contrasts, thereby limiting its use in treatments, illness tracking and clinical trials. MR also has some drawbacks, such as high cost and long exploration time, among others [11]. Motivated by all these drawbacks in the current techniques, during the last few years, a part of the scientific community has focused its efforts on the research and development of alternative techniques trying to overcome these limitations. One of these experimental techniques is based on medical image building by means of electromagnetic signals in the microwave frequency range. These systems bring a low-cost solution that is harmless for both the patient and the medical staff, and they feature interesting characteristics such as portability, fast operation and low energy consumption, among others. In comparison with conventional imaging systems, microwave systems do not require specific adaptation of the medical facilities (as it is required, for example, for CT or MR equipment), which opens the possibility to use these systems in outpatient scenarios or in isolated or lowly populated areas. Also, they can perform quasi-real-time operations. Due to these reasons, these systems are currently being proposed for different diagnosis and monitoring applications in the biomedical context [12].

Medical microwave imaging (MWI) systems implement a non-invasive technology usually working in the 1–10 GHz frequency range [13]. These systems are based on the detection and analysis of the reflections seen in microwave signals emitted by antennas when traveling through the different biological tissues of the organism. These reflections are due to the different dielectric properties between different tissues [14]. This is a non-ionizing technology that utilizes a very low emission power—more than 100 times lower than the power emitted by current mobile phones and roughly 100 times lower than the limits imposed by the SAR (Specific Absorption Rate) regulations, which all the electromagnetic emissions in biological contexts must comply with [15,16,17]—thus ensuring the full safety of these systems and the consequent possibility of using them repeatedly [18]. Currently, we can find several MWI devices and prototypes for different medical applications, including breast cancer detection [19,20,21], dynamic image building for cardiovascular systems [22], brain tumor classification [23], fast image building after cerebrovascular accident [24,25], or brain-shift detection during brain tumor surgery [18].

In most of these systems, the state of the art consists of proofs of concepts experimentally tested in laboratory conditions involving phantom materials mimicking the biological tissues [25,26,27,28]. However, there are certain advanced cases, especially those related to brain hemorrhage and breast cancer detection, that are now undergoing clinical trials in real contexts with real patients in pursuit of full validation of the technology [29,30,31,32,33]. In the specific case of aneurysm detection, there are published works related to MWI systems associated with brain aneurysms, thus providing considerable advancements for the detection of brain hemorrhages by this non-invasive medical imaging technique [34,35,36,37]. To the best of our knowledge, no studies are available on the application of MWI techniques for AAA detection, while the convenience of the use of this technology for similar contexts is being constantly acknowledged. It is, therefore, logical to expect that the same advantages of these systems that were found in other contexts could be of interest for the application of AAA detection.

In this work, we show a prototype of an MWI system based on radar techniques for AAA detection, which is devoted to demonstrating the potential of these techniques in this context. The device is composed of 16 independent identical monopole antennas placed on the same plane over a human torso phantom. The device also has a switching network implementing the sequential connection of the antennas with the measurement equipment and a control system run by a computer devoted to mastering and automatizing the operation of the system. This computer also runs the required signal processing and medical imaging algorithms. The system has been calibrated and fine-tuned to accurately detect and locate certain artifacts placed at different distances and positions within the torso phantom, emulating the presence of an AAA at different locations. The setup for the experimental assessment of the proposed system involves some simple but functional aorta and aneurysm phantoms in addition to the torso phantom. The AAA detection and location capabilities of this system are demonstrated through measurements with the aorta and aneurysm phantoms at different locations and the corresponding medical images obtained, as the system is able to effectively detect and locate the AAA in all cases.

The article is organized as follows: In Section 2, the hardware system is described, with a special focus on the antenna we developed for this application. Section 3 explains the signal processing and medical imaging algorithms in our system. Section 4 gives details on the calibration and fine-tuning process. The experimental evaluation and results for the proof-of-concept of the proposed system for AAA detection and location are presented in Section 5. Finally, the main conclusions of this work are gathered in Section 6.

## 2. Hardware System

**Figure 1:**
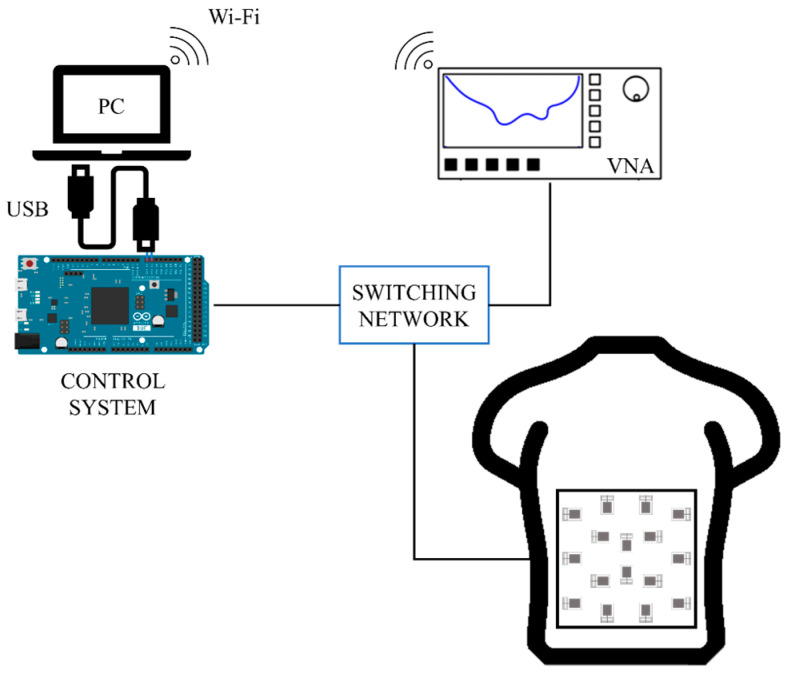
Connection diagram of the whole system, including the VNA, the control system, the switching network and the ensemble of the antennas.

The non-invasive microwave medical imaging system shown here is composed of 16 identical monopole-printed antennas like the one described in the following subsection. All these antennas are placed over a human plastic torso dummy, acting as a torso phantom. The system makes use of the Vector Network Analyzer (VNA) ZNLE6 from Rhode&Schwarz to measure the reflection parameters of the antennas. All this equipment is mastered through a control system and a switching network devoted to the proper sequential connection of each one of the antennas to the ports of the VNA. Figure 1 depicts a connection diagram of the whole system.

**Figure 2:**
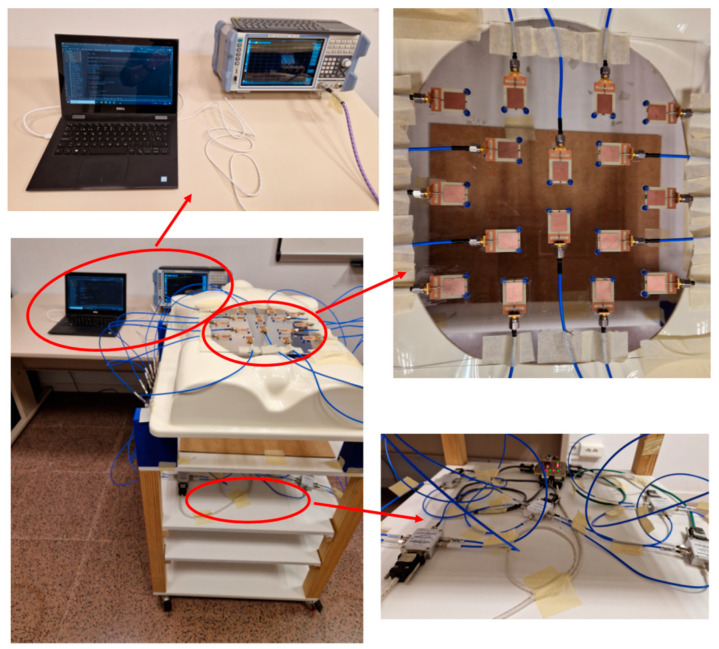
Hardware system, including the overall setup (**bottom left**), the control and measurement equipment (**top left**), the antennas (**top right**) and the switching elements (**bottom right**).

The control system and the switching network autonomously select the right antenna at each moment and connect it to the VNA for measurement. This is performed thanks to a computer running a Python script devoted to the management of the connections with the ports of the VNA and to sending the right commands to the VNA, and a microcontroller implementing the electronic management of the switching elements at the right time, thereby automating the whole measurement process. After gathering the measurements of all the antennas, the system keeps the reflection parameter associated with each antenna, and it then runs the signal processing and image generation algorithms in order to obtain the results. Some photographs of the hardware system involved in this work are shown in Figure 2, including detailed pictures of the control and measurement equipment, the switching elements and the antennas.

The overarching element in this system is the wideband antennas placed over a methacrylate sheet with 300 × 300 × 2 mm^3^ dimensions. This sheet is then placed over a torso phantom with a hollow in the measurement space, approximately corresponding to the stomach space in a human body. In this implementation, we used 16 independent antennas. The antennas were placed following a geometrical pattern-seeking to avoid the reflection coefficient of any antenna being affected by the presence of the neighbor antennas, i.e., to avoid any interferences or interactions between antennas. The minimum spacing between antennas to avoid these effects was found to be 25 mm by means of simulations. This placement pattern also seeks to cover all the measurement space in an equispaced manner. All the antennas were placed in orthogonal positions so that no boosted directions of detection were found in the system, and possible effects associated with the polarization of the antennas were avoided. Also, when placing the antennas, we considered that the main purpose of the system is the detection and evaluation of the aorta, which is always located in a longitudinal position inside the human torso. The final positioning of the antennas can be seen in Figure 2, where it can be appreciated that the torso phantom is located in a higher position than the workspace with the aim of easing the handling and measurement of the rest of the phantoms involved.

### 2.1. Antennas

**Figure 3:**
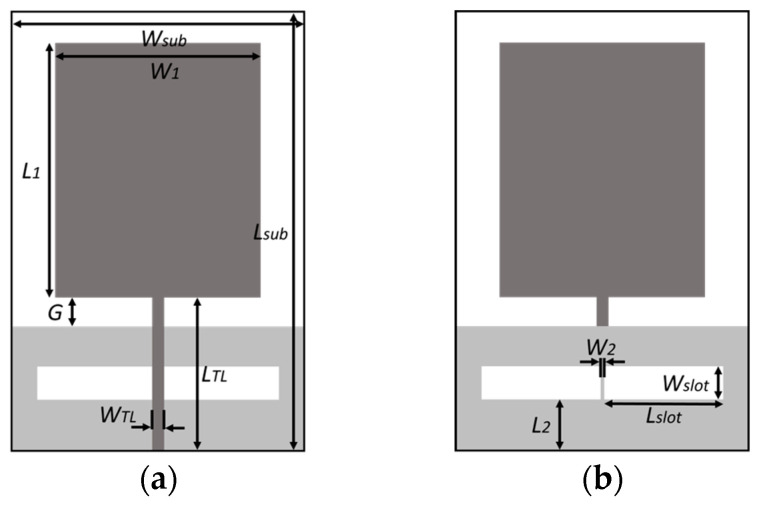
Design of the proposed antenna: (**a**) Top; (**b**) Bottom.

The proposed antenna is a monopole rectangular patch antenna with microstrip feed and two slots in the ground plane. We simulated and designed the antenna with the full-wave simulation software HFSS 2021 R2, considering a piece of low-loss, off-the-shelf FR4 substrate (Er = 4.4; tan d = 0.02) with a width of 0.4 mm. During the design process, starting from a conventional monopole rectangular patch antenna, we modified its shape with parametric simulations in order to achieve both the widest bandwidth and the lowest operating frequency possible. Then, two slots were etched in the ground plane with the aim of increasing the bandwidth, especially in the highest frequencies. Figure 3 shows the final design of the antenna and the description of its dimensions.

**Figure 4:**
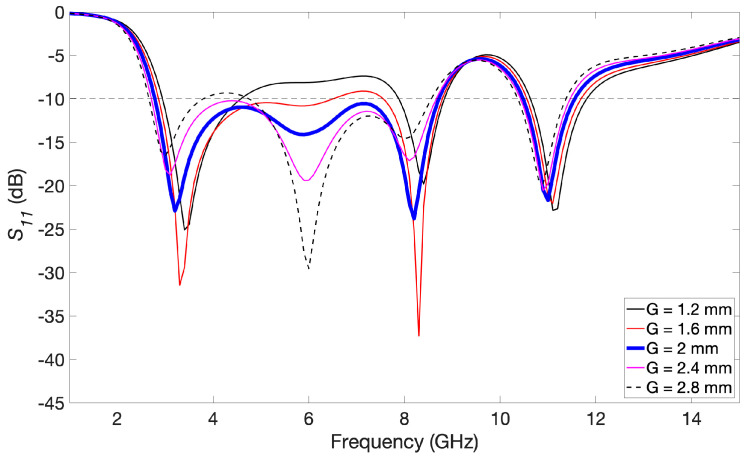
Reflection coefficient for several values of dimension *G*. The best result, highlighted with a wider blue line, was observed for *G* = 2.0 mm.

By parametric simulations, we studied the impact that each dimension has on the response of the antenna. Each dimension has been consequently set so that the lowest operating frequency possible and the widest bandwidth are obtained. As an example, Figure 4 plots the reflection coefficient (*S*_11_) of the simulated antenna while the value of dimension *G* (the distance between the radiating patch and the ground plane) is modified. It can be easily seen that this dimension has a remarkable impact on the characteristics of the antenna, with the best behavior being found for case *G* = 2.0 mm (the corresponding curve is highlighted with a wider blue line). After carrying out this optimization process for all the dimensions by simulations, we obtained a bandwidth ranging from 2.8 GHz to 8.7 GHz, which means a bandwidth of roughly 6 GHz for a reflection coefficient lower than −10 dB. It should also be noted that another operating frequency band of more than 1 GHz at higher frequencies is available, from 10.5 GHz to 11.6 GHz. The final chosen dimensions of the proposed antenna are listed in Table 1.

**Figure 5:**
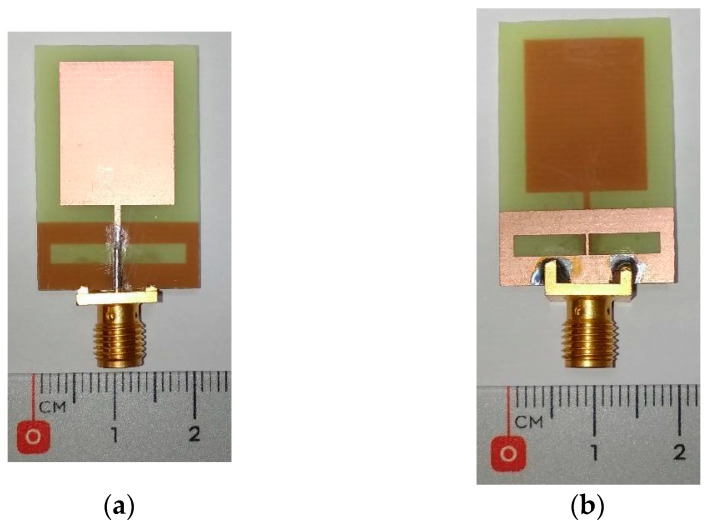
Photographs of the fabricated antenna: (**a**) Top; (**b**) Bottom.

**Figure 6:**
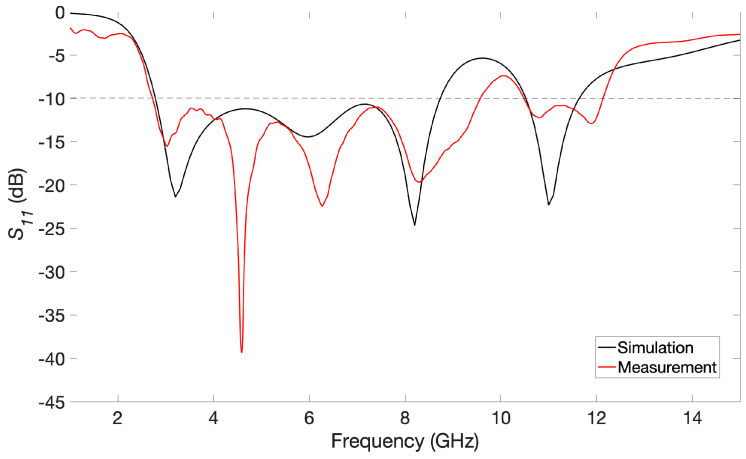
Simulated and measured reflection coefficients for the proposed antenna.

The proposed antenna was fabricated by means of photolithography and chemical attack over the two-sided copper-clad FR4 substrate. Some photographs of the fabricated prototype, with a final size of 20 × 30 mm^2^, can be seen in Figure 5. The behavior of the antenna has been assessed in an anechoic chamber with the Vector Network Analyzer (VNA) E8363B from Agilent Technologies. Figure 6 plots both the simulated and experimentally measured reflection coefficients. A good agreement between both curves can be seen, thus validating the optimization and fabrication process of the antenna. The experimental operating frequency range considering *S*_11_ < −10 dB is found to be between 2.7 and 9.6 GHz, which yields an experimental bandwidth of 6.9 GHz.

**Figure 7:**
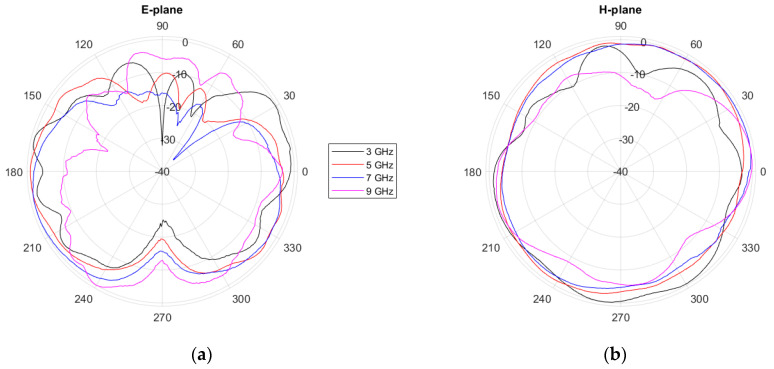
Experimentally measured normalized radiation patterns for the proposed antenna: (**a**) *E*-plane; (**b**) *H*-plane.

The radiation patterns for the proposed antenna were also characterized inside the anechoic chamber using a normalized horn antenna as a reference. Figure 7 shows these experimental results for the two main planes of the antenna (*E*- and *H*-plane) and for the most significant frequencies within the operating bandwidth. As can be seen, these radiation patterns entail an omnidirectional behavior of the antenna, as usual for monopole antennas. The simulated radiation patterns (not shown for the sake of brevity) show a similar response to the experimental ones. The experimental gain of the antenna ranges between 1.4 and 3.0 dBi within the operating frequency range.

The envisioned application for the designed antenna in this work is based on the transmission and reception of signals involving a considerable bandwidth. Due to this reason, we performed an analysis of the transmission capability of this antenna when wideband time pulses are involved, focusing also on the dispersion and integrity of the transmitted pulses. The setup for this experiment was composed of two identical antennas standing face-to-face inside the anechoic chamber, 30 cm apart from each other. It should be noted that this is enough distance to ensure far-field transmission. We obtained the frequency-domain transmission parameter (*S*_21_), both with simulations and experimental measurement; after proper processing, we obtained the time-domain transmitted and received signals following the procedure described in [13].

**Figure 8:**
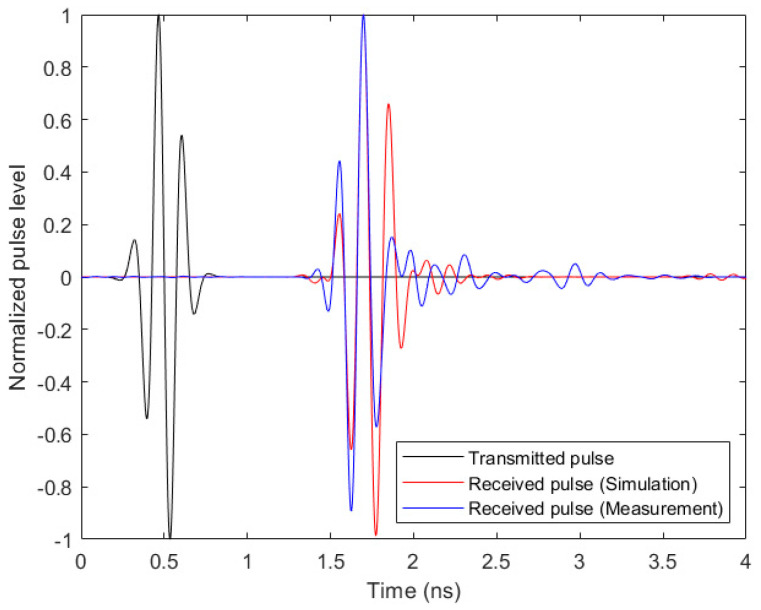
Transmitted pulse and received pulses in simulation and measurement for the analysis of the transmission capability of the antennas and the distortion.

In Figure 8, a comparison between the time-domain transmitted and received pulses by the antenna can be seen for both simulation and measurement. It is easy to note that the received pulses in simulation and measurement are remarkably similar to each other, as well as to the transmitted one. From the phase of the *S*_21_ parameter, the group delay in the system can be calculated. We obtained fairly similar and almost constant group delays over the whole antenna bandwidth in both simulations and measurements. The average delay is 0.13 ns in simulation and 0.11 ns in experimental measurement.

Furthermore, the System Fidelity Factor (SFF) is a useful metric for quantifying how much the transmitted pulse is affected by the antenna system. The SFF numerically computes the similarity of the transmitted and received pulses by means of a correlation function by computing the ratio between the energy of the convolution of the two pulses and the overall energy of each one of them separately [13,38]. Following this procedure, we obtained an SFF of 97.5% in simulations and an SFF of 96.3% in experimental measurements. These data reinforce the statement that the transmitted and received pulses are notably similar to each other, which allows us to expect high integrity of the signals transmitted by the antennas. Consequently, all these results lead to the conclusion that the proposed antenna is adequate for use in microwave medical imaging systems based on the transmission of wideband pulses.

### 2.2. Switching and Electronic Control System

All the antennas are connected to the switching network. This network includes five RF off-the-shelf switches (ref. ZSWA4-63DR+ from Mini-Circuits^®^, Brooklyn, NY, USA). Each switch has four 50 Ω-matched outputs working in the 10 MHz–6 GHz range. They also feature high switching speed (320 ns, according to the provider) and low losses. The maximum loss for the RF system, including switches and wires, is 7.3 dB at 6 GHz. This network sequentially connects each one of the antennas to port 1 in the VNA, ensuring that at each moment, there is only one single antenna transmitting/receiving. The switches are connected to the control and powering subsystem, managed by the microcontroller, through DB9 cables. The powering is straightforwardly obtained from the 5 V voltage provided by the USB port of the computer. This voltage is immediately tuned to 3.3 V (the required voltage for the switches) by means of a DC/DC converter and then driven to power the switches. The maximum current consumption of the whole switching network is lower than 2 mA.

The control system is mastered by an Arduino Due device, which includes an AT91SAM3X8E (Atmel Corporation, San Jose, CA, USA) microcontroller. This device implements a truth table engineered to activate the right control inputs of the switches at each moment so that the right antenna is effectively connected to the VNA through the switching network at the right time. Part of the control system and the switching network are located at a lower level with respect to the torso phantom, trying to avoid as much as possible the interferences in the signals received by the antennas. In this work, this system is connected to a single port in the VNA, which is set to make measurements from 10 MHz to 6 GHz with 5001 frequency points, an intermediate frequency filter of 10 KHz and an emission power in the VNA of 0 dBm (1 mW). The radiating power in the antennas, considering the RF system losses and the radiation efficiency of the antenna, is higher than –7.8 dBm. It should be noted that this emission power is sufficiently low so that the proposed system can be deemed harmless to the users [17,20].

We analyzed the signal-to-noise ratio (SNR) of the system by means of a normalized calibration standard, computing the noise spectral power and following the method described in [20,39]. The signal received by the antennas was over −30 dBm (for an emission power of 0 dBm) in all cases, whilst the noise power in the system was found to be lower than −58 dBm. The SNR is, therefore, +28 dB. Furthermore, as mentioned above, this system is mastered by a computer, which is also connected to the VNA through a WLAN network. This allows the computer to load the electronic calibration previously made for each one of the antennas in the VNA at the right moment and to receive and store the measured reflection parameter (*S*_11_) for each antenna. The computer later processes all these responses to obtain the time-domain reflections seen by each antenna and to build the corresponding medical image. The whole measurement process takes less than 1 min.

## 3. Image Generation and Processing

After the measurement of the scattering parameters of all the antennas is completed, the responses of the antennas are processed. The system is intended to generate the reflection signals received by each antenna and to build an image representing the bodies, objects or targets detected by the antennas. With this purpose, after transmission of the pulse, it is important to identify the echoes reflected back to the antennas by these objects. By assessing the time of flight of each echo to each antenna, the position of the objects can be determined. However, the echoes are not so simple to identify since they are composed of a superposition of multiple reflections coming not only from the target but also from other elements or objects in the surroundings, such as antennas, wires, electronic components, etc. The resulting signals are remarkably complex, and the analysis could become prohibitively cumbersome.

Under these circumstances, the processing of the responses of the antennas can be notably simplified by applying a similar technique to the one described in [18], assuming a linear model and applying the superposition principle. Firstly, a reference measurement is made with each antenna in an empty setup, with no elements in the measurement space. The resulting signals are a linear combination of the undesired echoes (i.e., the echoes not associated with the targets) in the system. Secondly, a new measurement is made with the antennas when the objects or bodies are in the measurement space. Now, the recorded signals will be a linear combination of the undesired echoes and the echoes coming from the targets. Then, by subtracting the first signal (empty setup) from the second signal (setup with targets) for each antenna, a third signal, including only the echoes associated with the targets, is obtained. Finally, the time of flight associated with the targets is obtained as the local maxima of the cross-correlation between this third signal and the transmitted pulse.

It should be highlighted that this technique is based on certain assumptions and, consequently, has some limitations. For example, the dispersion of the signals has not been considered. This phenomenon makes the pulses reflected back slightly different in shape and frequency content than the transmitted pulses (in addition to delay and attenuation). These differences will hinder the total removal of the undesired echoes, and the linearity condition will not be strictly met. This and other disturbing circumstances (e.g., slight movements of the setup) will unavoidably add electronic and quantization noise to the measurements. However, as discussed in [18], the distances in our system are relatively short considering the speed of the wave. The waves propagate through free air in our setup, and the dispersion is meaningless for the undesired echoes, chiefly coming from small plastic or metallic parts. Therefore, the aggregate effect of these disturbing elements can be considered as Gaussian additive noise that will have an impact on the precision of the system but not on its functionality.

Before obtaining the difference signals, which contain the information on the reflections of the objects to be evaluated, a certain pre-processing is applied with the aim of removing noise and unwanted artifacts. The most remarkable is the use of the inverse chirp-Z transform, which allows a very good reconstruction of the signals in the time domain [40,41], and the Hilbert transform, which allows the elimination of most of the noise since the interesting information is usually located in the middle frequencies of the signal spectrum [20]. We finally make the subtraction to remove the undesired echoes, as explained before.

When the signals of the reflections of the antennas have been effectively obtained, two beamformer algorithms can be used for medical image generation: Delay-and-Sum (DAS) algorithm and the Improved Delay-and-Sum (IDAS) algorithm. These algorithms are widely used in applications related to the detection of biological markers by means of microwave signals [42], and they provide detection and positioning capabilities for the objects or artifacts showing a certain dielectric dispersion of the microwave signals. Both algorithms rely on a mesh or spatial distribution of the problem under analysis followed by a computation, for each one of the points in the mesh and for each antenna, of the time delay in the signal, assuming that the transmission between the antenna and each point happens through a straight line. In the DAS algorithm, the obtained responses are summed, squared and integrated into a predefined time window. This process yields a qualitative intensity value for the signal associated with the point under analysis [43]. Formally:

(1) $$I\left(r_{0}\right)=\int_{0}^{T_{Win}}{\left[\sum_{m=1}^{M}x_{m}\left(\tau_{m}(r_{0})\right)\right]}^{2}dt,$$

(2) $$\tau_{m}(r_{0})=\frac{d_{m}}{v}f_{s},$$

(3) $$v=\frac{c}{\sqrt{\varepsilon_{r}}},$$

where *T_Win_* is the predefined time window, *r*_0_ is the synthetic focal point under computation, *x_m_* is the backscattered radar signal recorded at the *m*th antenna, *d_m_* is the round-trip distance from the *m*th transmitting antenna to the point *r*_0_, *f_s_* is the sampling frequency, *ε_r_* is the real part of the relative dielectric permittivity of the medium through which the signal is traveling, *v* is the average speed of propagation of the waves in the medium, and *c* is the speed of light in vacuum.

The IDAS algorithm includes a Coherence Factor (CF) in the equation for the computation of the intensity of the reflection used in the DAS algorithm, as defined in (1). This factor is considered as a weighting factor for correction of the intensity of the signal in each one of the computation points. The CF takes higher values in the points where the reflections are due to different values of the dielectric permittivity, which provides more accurate detection and location capabilities for the artifacts in the scenario. IDAS equation is as follows [43]:

(4) $$I\left(r_{0}\right)=CF(r_{0})⊙\int_{0}^{T_{Win}}{\left[\sum_{m=1}^{M}x_{m}\left(\tau_{m}(r_{0})\right)\right]}^{2}dt,$$

(5) $$CF(r_{0})=\frac{{\left|\sum_{m=1}^{M}x_{m}\left(\tau_{m}(r_{0})\right)\right|}^{2}}{{\sum_{m=1}^{M}\left|x_{m}\left(\tau_{m}(r_{0})\right)\right|}^{2}}$$

The time required to process the signals and generate the corresponding images with both algorithms in this system is shorter than 30 s with a 12th generation I7-1255U processor (Intel Corporation, Santa Clara, CA, USA) and 16 GB RAM memory. With this setup, the total time required in this system to perform the measurement, process it and generate the final images is approximately 90 s, which is remarkably shorter than the usual times required by the current medical imaging methods used in these circumstances, such as CT or MR.

## 4. Calibration and Fine-Tuning

### 4.1. Calibration of Measurements at Planes Parallel to the Antennas

Calibration and fine-tuning are essential for the proper functioning of any electronic instrument, and they are even more crucial for medical imaging systems. In this sense, it is important to test the detection capabilities of the proposed system with known objects and positions, as well as to adjust all the necessary parameters accordingly in order to achieve the best detection capabilities. With this purpose, we performed a two-stage calibration strategy. Firstly, an electronic calibration for each antenna allows the placement of the measurement plane at the antenna connector, thus eliminating the influence of the switching system and connecting cables used in the system. Secondly, copper-clad RF substrate sheets were used at known distances from the antenna plane. Wide enough sheets (350 × 250 mm^2^) were selected in order to cover all the measurement areas, ensuring good reflections for all the antennas at the known distances.

**Figure 9:**
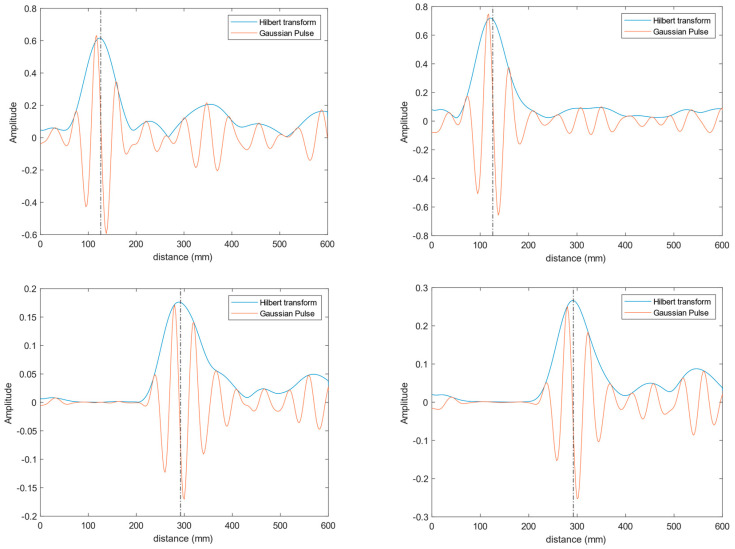
Time-domain signals received by antennas #9 (**left column**) and #14 (**right column**) when the copper-clad sheet was put at 126 mm (**top row**) and 292 mm (**bottom row**). In the plots, the reflection detected by each antenna (orange line), its Hilbert transform (blue line) and the detected distance (dotted black line) can be seen.

**Figure 10:**
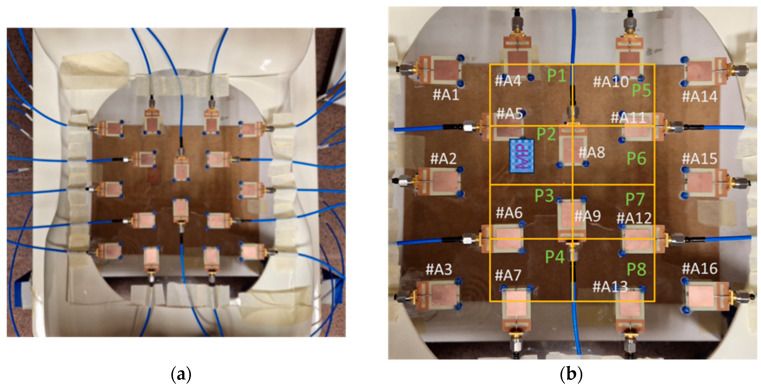
Pictures of the grid over the torso phantom: (**a**) Picture of the measurement grid; (**b**) Scheme of the positioning and labeling of the different antennas, the grid and the copper-clad sheet (labeled MP).

Once all the signals received by the antennas have been gathered, they are transformed to the time domain by means of the procedure described in Section 3. Figure 9 shows an example of the time-domain signals obtained for antennas #9 and #14 (see Figure 10 when the copper-clad sheet is put at 126 mm and 292 mm from the antennas. The results for these antennas are shown because they are representative of the different areas of the torso phantom: antenna #9 is in a central position, whilst antenna #14 is located in one of the corners. As can be seen, the Hilbert transform of the time-domain response of the antenna provides us with an accurate determination of the distance to the copper-clad sheet by just obtaining the distance associated with the maximum of the signal.

**Figure 11:**
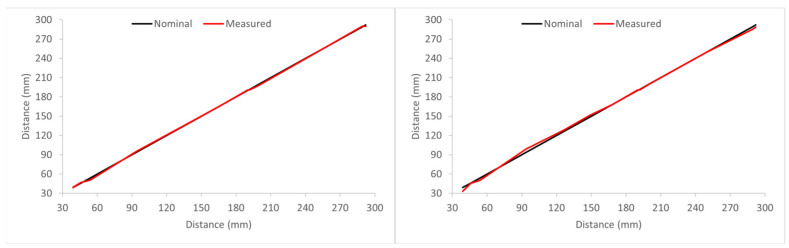
Detected distances (red line) to the copper-clad sheet by analyzing the reflections measured in antennas #9 (**left**) and #14 (**right**) for the different distances compared to the real distances (black line).

We repeated this process by placing the copper-clad sheet several distances from the antennas. Figure 11 shows a comparison between the distances at which the sheet was placed, ranging from 40 to 292 mm, and the detected distance with this procedure by antennas #9 and #14. It can be seen that in both cases, the agreement is good, and the error in the determination of the distance to the sheet is considerably low, specifically lower than 3.8%.

### 4.2. Calibration of Measurements at Different Positions within a Plane

After calibrating the detection of distances, which allows us to ensure that the distance at which the objects are detected is appropriate, we conducted an experimental study of the capabilities of our system to position smaller objects within the operation scenario. To that end, we set a grid over the scenario, thereby dividing the measurement area into 2 × 4 spatial positions. We made several measurements, placing a smaller copper-clad sheet of 55 × 40 mm^2^ at different positions within the grid. The pictures in Figure 10 show the grid over the torso phantom. This grid is placed at 238 mm under the antennas, becoming the reference plane. In the example in Figure 10, a small copper-clad sheet (shown as MP) was placed in the P2 position.

**Figure 12:**
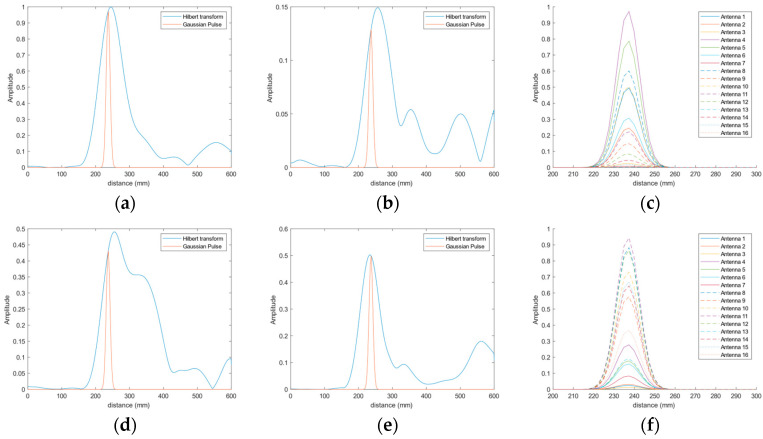
Time-domain signals involved in the process of detecting the calibration copper-clad object placed at positions P1 (**top row**) and P6 (**bottom row**) on the reference plane: (**a**) Time-domain signal for antenna #4 (blue line) and generated narrow pulse at 238 mm distance (orange line) for the object in P1; (**b**) Time-domain signal for antenna #11 (blue line) and generated narrow pulse at 238 mm distance (orange line) for the object in P1; (**c**) Generated narrow pulses for all the antennas for the object in P1; (**d**) Time-domain signal for antenna #4 (blue line) and generated narrow pulse at 238 mm distance (orange line) for the object in P6; (**e**) Time-domain signal for antenna #11 (blue line) and generated narrow pulse at 238 mm distance (orange line) for the object in P6; (**f**) Generated narrow pulses for all the antennas for the object in P6.

The detection of the position of the object entails the measurement of the time-domain response of each one of the antennas and the computation of their Hilbert transforms, as discussed in the prior section. In this case, aiming at the detection of the objects within a plane at a fixed and known distance to the antennas (238 mm in the case under analysis), we obtained the amplitude, measured at the distance to the measurement plane, in the response of each antenna. Then, we generated new narrower pulses (14 ns long, considering the usual full width at half maximum, FWHM) at the same amplitudes and fixed distance for each antenna. These pulses contain the information on the reflection received by each antenna at a fixed distance. Figure 12 shows this process for the placement of the object at two different positions and for two antennas.

Particularly, Figure 12a plots the received signal by antenna #4 when the object is placed at position P1 (see Figure 10), i.e., under antenna #4. The blue line is associated with the Hilbert transform obtained from the reflections for all the distances, whereas the orange line corresponds to the narrower pulse generated with the obtained amplitude at 238 mm distance. Figure 12b shows the same process but for antenna #11, which is far from position P1. It can be observed that, in this case, the maximum amplitude of the signal received by the antenna is located at a greater distance since the object is further from this antenna. This implies that, at the fixed distance of the reference plane (238 mm), the amplitude of the reflection is lower; therefore, the newly generated pulse has a lower amplitude than that from antenna #4.

Figure 12c gathers the pulses that were generated with the information from all the antennas for the object placed at position P1. It is straightforward to note that the antennas receiving the most power from the objects placed in the reference plane are the antennas that are closest to the position of the object under analysis. In this case, these antennas (in descending order of received power) are #4, #1, #5, #8 and #10. Finally, Figure 12d–f show a similar analysis for the case of the object placed at position P6, just under antenna #11. Consequently, it is indeed antenna #11 that receives the highest power in the reflection associated with the copper-clad object on the reference plane, and it is, therefore, the pulse associated with this antenna the one with the greatest amplitude (see Figure 12f).

**Figure 13:**
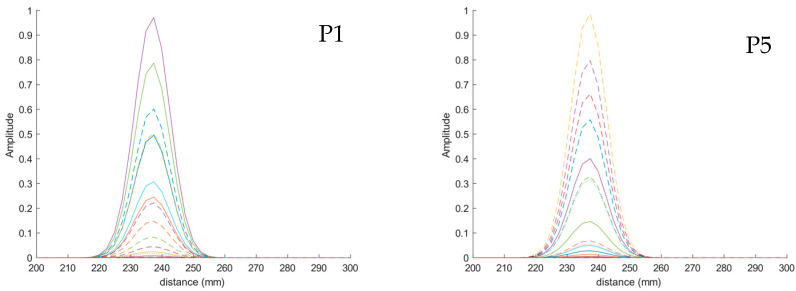

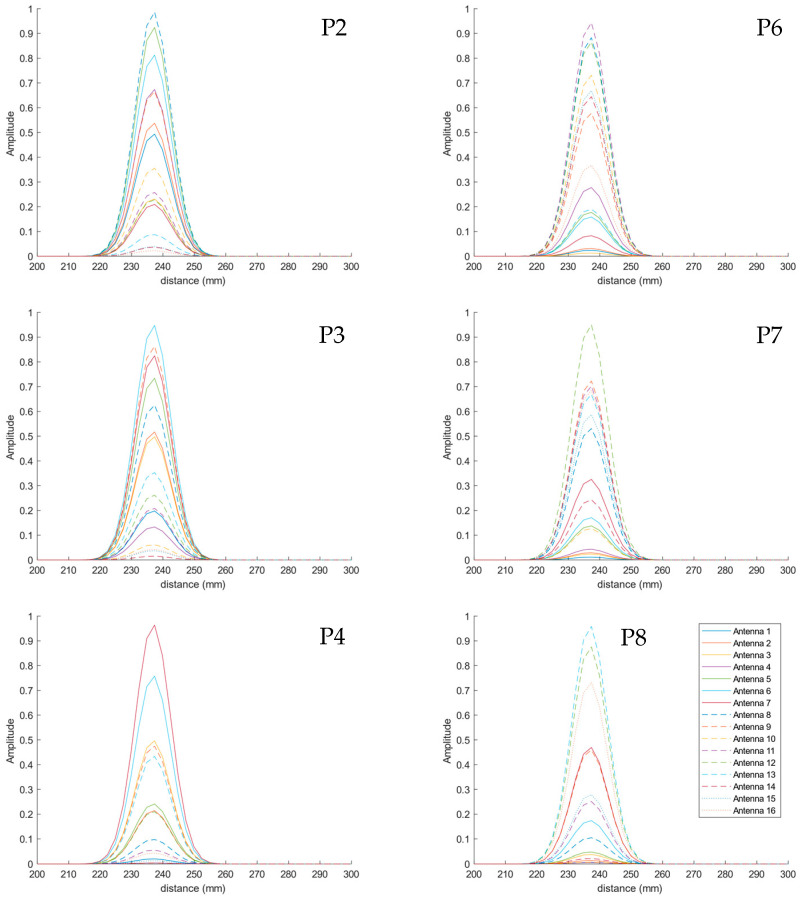
Time-domain signals associated with the reflections received by each one of the antennas in the 8 positions of the grid under analysis. The time pulses have a FWHM of 14 ns.

The analysis shown in Figure 12 has been repeated for all the positions in the grid. The results show that in all the cases, the antenna receiving the highest power at the point corresponding to the reference plane (238 mm) is the one placed just over the position of the object. In Figure 13, the generated pulses for each antenna in the reference plane for each position in the grid are shown. As can be seen, in all the positions in the grid, we verified that the closest antennas are the ones receiving the highest power, thereby proving the potential of the system for detecting and locating artifacts on a reference plane.

## 5. Experimental Validation and Proof-of-Concept

### 5.1. Experimental Setup

**Figure 14:**
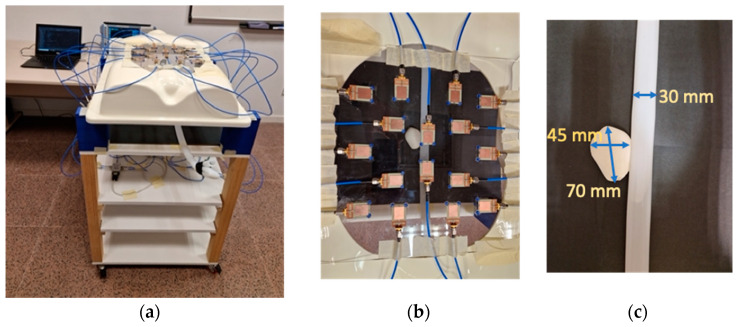
Photographs of the setup for the experimental proof-of-concept as to the measurement of aorta and aneurysm phantoms: (**a**) Overview of the full setup; (**b**) Detailed picture of the placement of the aorta and aneurysm phantoms with respect to the antennas for a given measurement; (**c**) Zoom-in of the aorta and aneurysm phantoms, including their dimensions.

Having proved the capability of the system to detect and locate objects placed at different distances and planes, we made a proof-of-concept devoted to the assessment of the detection functionality of the system in more complex scenarios, such as the one emulating the detection of an abdominal aortic aneurysm. The pictures in Figure 14 show the experimental setup for such a proof of concept. Inside the torso phantom, we included an object acting as a healthy aorta phantom: a plastic tube with a length of 600 mm and a diameter of 30 mm, filled with water. We used water since blood has a remarkably high water content, thus emulating the dielectric permittivity of blood, while water is much easier to obtain and manipulate. For an aneurysm phantom, we used a balloon filled with water as well, with dimensions of 70 × 45 × 45 mm^3^ (see Figure 14c). We placed it next to the aorta phantom, touching it. Both the aorta and the aneurysm phantoms were placed at different locations within the measurement grid, always in a plane at 210 mm from the antennas (the current reference plane).

In this more complex scenario involving phantoms with biological material and with relatively big sizes with respect to the measurement area for the antennas (which means that some antennas will receive important signals), we used the medical imaging algorithms DAS and IDAS, as described above. These are two of the commonly used imaging algorithms in biological tissue detection by means of microwave antennas [42], and they were proved to be convenient for determining the contribution of each antenna in complex scenarios [44,45]. As a novelty, in this work, considering that the evaluation of the contributions of the antennas has to be conducted at a given distance from the antennas (in the plane where the objects under analysis are placed), we employed a modified version of these algorithms in which the information provided by each one of the antennas is obtained by means of the process described in Section 4. As mentioned above, the time-domain response of the received reflection is obtained with the Hilbert transform for each antenna. Then, from this response, the contribution of the antenna for the distance to the object (the reference plane) is evaluated, and a new narrow pulse of 14 ns FWHM is generated for each antenna. These pulses are then fed into the imaging algorithms.

### 5.2. Proof-of-Concept

At an initial stage, considering only the healthy aorta phantom, with no aneurysm, three measurements were made, placing the aorta phantom in three different positions within the measurement grid (namely on the right side, in the middle and on the left side). The purpose of these measurements was to assess the capability of the system to detect and locate the aorta phantom before evolving into more complex measurements. The DAS algorithm was used in this case for image generation since it was proved to be appropriate for representing objects with relatively big sizes, such as our aorta phantom [44,46]. In all the cases, the measurements were set at a distance of 210 mm from the antennas, which corresponds to the position of the plane where the aorta phantom was placed.

**Figure 15:**
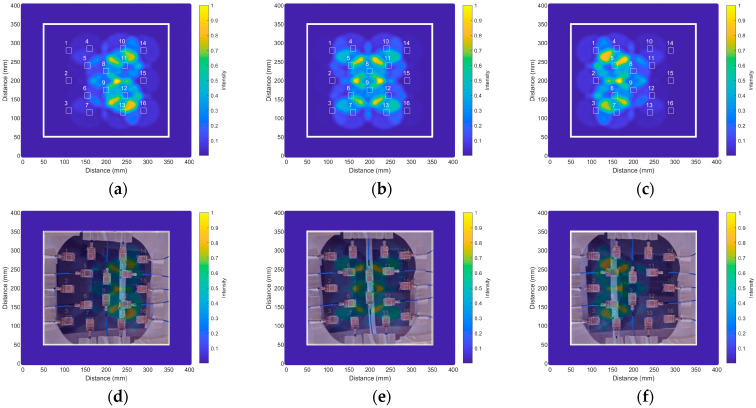
Results obtained with the modified version of the DAS algorithm for the detection of the aorta phantom in three different positions: (**a**) Result for the aorta phantom on the right side of the measurement area (the numbers refer to the corresponding antennas); (**b**) Result for the aorta phantom in the middle of the measurement area; (**c**) Result for the aorta phantom on the left side of the measurement area; (**d**) Result for the aorta phantom on the right side superimposed on a photograph of the real setup; (**e**) Result for the aorta phantom in the middle superimposed on a photograph of the real setup; (**f**) Result for the aorta phantom on the left superimposed on a photograph of the real setup.

Figure 15 shows the results for the three cases. Specifically, Figure 15a shows the resulting medical image associated with the aorta phantom placed on the right side of the measurement area. The contribution of each antenna (shown as a circumference around the antennas), the sum of the contributions of all antennas provided by the DAS algorithm, and how the system detects an elongated object in the column made of antennas #10, #11, #12 and #13 (the antennas are depicted in the images with white rectangles with the dimensions of their radiating patches), which are the closest ones to the aorta phantom in this case, can be seen. For better visualization and understanding, in Figure 15d, we superimposed this result on a photograph of the actual position of the aorta phantom, taken from the upper space of the antennas. It is easy to notice the good agreement between the results for our modified version of the DAS algorithm and the real scenario.

This analysis has been repeated for the two remaining positions of the aorta phantom. Figure 15b,e show the result when the aorta phantom was placed in the middle of the measurement area, just under antennas #8 and #9, while Figure 15c,f show the result for the case of the aorta phantom placed on the left side of the measurement area, i.e., under antennas #4, #5, #6 and #7. In all cases, we observed that the proposed medical imaging algorithm is useful for the right detection and location of the aorta phantom inside the torso phantom.

The next step in the proof of concept consisted of assessing the capability of the system to detect a deformation of the aorta, which is associated with an AAA. The new setup involved the aorta phantom used in the previous measurements in addition to a balloon filled with water, acting as a phantom for the deformation of the aorta due to the AAA (as shown in Figure 14c. In this case, the modified version of the IDAS algorithm was preferred for medical image generation due to its better accuracy for the detection of anomalous, localized objects [44,46]. The reference plane was set, this time to the intermediate point of the aneurysm phantom, found at 180 mm from the antennas.

**Figure 16:**
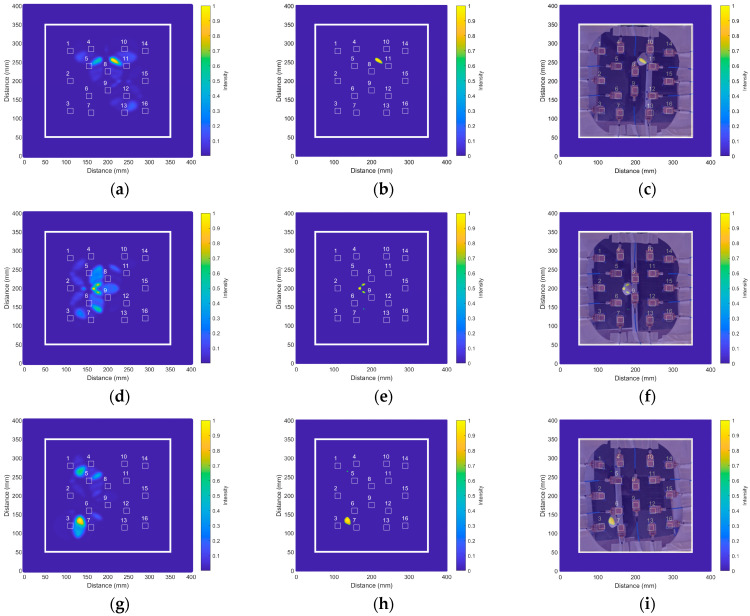
Aneurysm detection results for three different positions by means of the modified version of the IDAS algorithm: (**a**) Result associated with the aorta phantom placed on the right side of the measurement area and the aneurysm in the upper region (the numbers refer to the corresponding antennas); (**b**) Result from (**a**) after applying the threshold of 0.6 units of intensity; (**c**) Result from (**b**) superimposed on a photograph showing the real positions of the aorta and aneurysm phantoms; (**d**) Result associated with the aorta phantom placed in the middle of the measurement area and the aneurysm in the middle region; (**e**) Result from (**d**) after applying the threshold of 0.6 units of intensity; (**f**) Result from (**e**) superimposed on a photograph showing the real positions of the aorta and aneurysm phantoms; (**g**) Result associated with the aorta phantom placed on the left side of the measurement area and the aneurysm in the lower region; (**h**) Result from (**g**) after applying the threshold of 0.6 units of intensity; (**i**) Result from (**h**) superimposed on a photograph showing the real positions of the aorta and aneurysm phantoms.

With this setup, we analyzed nine different experimental cases with different configurations of the aorta and the aneurysm phantoms within the measurement area. As an example, Figure 16 shows three of these cases, for which the aorta was placed in the three previous positions, and the aneurysm is placed at three different positions next to the aorta: in the upper region, in the middle region and in the lower region. Figure 16a shows the result for the aorta phantom placed on the right side and the aneurysm in the upper region (case 1 in Table 2, see below). As can be observed, the system effectively detects the anomaly (associated with an AAA) between antennas #8 and #11.

For better locating the detected artifact, the results were thresholded so that all the values in the image smaller than a certain threshold were removed. This threshold was experimentally adjusted for the current setup to 0.6, then kept constant for all the cases. The result after thresholding for the case of the aorta phantom placed on the right side with the aneurysm in the upper region is shown in Figure 16b. Finally, with the purpose of checking that the detected position of the aneurysm is in good agreement with the real position of the aneurysm phantom, the result (after thresholding) is superimposed on a photograph of the actual setup, taken from the upper space of the antennas (Figure 16c). As can be seen, the system detects and locates the aneurysm phantom with high accuracy.

Figure 16d–f show the results for the case of the aorta phantom placed in the middle of the measurement area and the aneurysm phantom placed also in the middle region, between antennas #5, #6, #8 and #9 (case 5 in Table 2). It can be seen that the algorithm also detects an artifact in this case. Once the result is thresholded and superimposed on the photograph of the actual scenario, we can observe a good agreement between the detected object and the location of the aneurysm phantom again. A similar result was found in the third case, as shown in Figure 16g–i, in which the aorta phantom was placed on the left side of the measurement area and the aneurysm phantom was placed in the lower region, between antennas #3, #6 and #7 (case 9 in Table 2). We obtained similar results for the rest of the measurements in the remaining 6 cases, which are not shown here for the sake of brevity.

The penetration depth (*PD*) can be obtained from the wavelength in vacuum and the dielectric properties of the materials the waves travel through [47]. In this case, considering the average dielectric properties of the aorta and an aneurysm [48], which are mainly composed of blood, the *PD* in the proposed system varies between 18.8 mm at 1 GHz and 3.2 mm at the highest frequency, 6 GHz. Also, the spatial resolution at this maximum frequency, computed as described in [49], is 3.5 mm. It can be seen that one of the benefits of wideband microwave imaging systems like the one proposed here is the potential to attain both good penetration depth thanks to the lowest frequencies and good spatial resolution thanks to the highest frequencies in the band.

Finally, for a better assessment of the results, we computed the experimental positioning errors for the AAA for the nine cases under study. To do that, as mentioned above, we applied the IDAS algorithm for generating the images, and we later applied the 0.6 threshold (the same procedure as the one shown in Figure 16). With the generated images, we obtained the coordinates of the centroid for each detected AAA, having the same origin of coordinates as in Figure 16. The real positions of the AAA phantoms were computed from their corresponding photographs for comparison (it should be noted that these data are prone to a certain error due to the manual positioning of the object). The results are shown in Table 2. In general, the errors are considerably low, with errors below 10 mm and relative errors below 5% in most cases.

## 6. Conclusions

In this work, we showed a microwave medical imaging system devoted to the detection of abdominal aortic aneurysms by means of 16 twin antennas placed on the same plane. The design of the antenna was optimized to achieve a wide bandwidth while remaining within acceptable dimensions for the antenna. A hardware system was implemented for automated sequential measurement of the antennas. We carried out a scrupulous calibration and fine-tuning of the system. A signal processing and image generation strategy was devised by adapting to our system-modified versions of DAS and IDAS algorithms for measurement in plane scenarios, leveraging the good performance of the DAS algorithm for detecting objects of considerable dimensions (such as the aorta phantom) and the precision brought by IDAS algorithm for the location of relatively small objects (such as the AAA phantom). We made a proof-of-concept of the proposed technology with a bespoke experimental setup composed of a human torso phantom housing an aorta phantom and an aneurysm phantom. The performance of the system was evaluated with several configurations of the aorta and the aneurysm phantoms within the torso phantom. The results showed good detection and location capabilities, as well as easy visual identification of the aneurysm phantom in the generated images. The assessment of the errors showed accurate detection with acceptable errors for the proposed application. This technology could be an interesting option for fast detection of aneurysms, avoiding the high costs, prolonged operation and risky use associated with the conventional equipment. Considering the results, we conclude that MWI technology may be a good option to take into account as a complement to the current medical imaging systems for AAA detection, thus bringing a low-cost solution suitable for outpatient use and harmless for both patients and medical staff.

## Figures and Tables

**Table 1:** Final dimensions of the proposed antenna. All data are in mm.

Parameter	Value	Parameter	Value	Parameter	Value
*W_sub_*	20.0	*W*_2_	0.3	*L_TL_*	10.5
*L_sub_*	30.0	*L*_2_	3.5	*W_slot_*	2.3
*W*_1_	14.0	*G*	2.0	*L_slot_*	8.1
*L*_1_	17.4	*W_TL_*	0.7		

**Table 2:** Positioning errors for the 9 experimental cases under study. All positions and absolute errors in mm. Pos. = Real position; Obt. = Obtained position.

AAA Case	Real Position		IDAS Obtained Position		Positioning Error			
	Pos. *x*	Pos. *y*	Obt. *x*	Obt. *y*	Error *x*	Rel. Error *x*	Error *y*	Rel. Error *y*
1	221.5	256.8	216.8	256.0	4.7	2.1%	0.8	0.3%
2	213.7	184.0	227.8	197.3	14.1	6.6%	13.3	7.2%
3	216.8	140.0	218.4	143.2	1.6	0.7%	3.2	2.3%
4	173.2	264.6	177.7	253.6	4.5	2.6%	11.0	4.2%
5	180.8	201.2	172.2	199.6	8.6	4.8%	1.6	0.8%
6	176.1	137.1	180.8	143.2	4.7	2.7%	6.1	4.4%
7	140.9	259.1	136.1	261.4	4.8	3.4%	2.3	0.9%
8	137.0	185.5	133.1	180.8	3.9	2.8%	4.7	2.5%
9	140.1	135.9	136.2	134.6	3.9	2.8%	1.3	1.0%

## Data Availability

Data are contained within the article.
